# Fecal microbiota and inflammatory and antioxidant status of obese and lean dogs, and the effect of caloric restriction

**DOI:** 10.3389/fmicb.2022.1050474

**Published:** 2023-01-12

**Authors:** Carla Giuditta Vecchiato, Stefania Golinelli, Carlo Pinna, Rachel Pilla, Jan S. Suchodolski, Asta Tvarijonaviciute, Camila Peres Rubio, Elisa Dorato, Costanza Delsante, Claudio Stefanelli, Elena Pagani, Federico Fracassi, Giacomo Biagi

**Affiliations:** ^1^Department of Veterinary Medical Sciences, University of Bologna, Bologna, Italy; ^2^Gastrointestinal Laboratory, Texas A&M University, College Station, TX, United States; ^3^Interdisciplinary Laboratory of Clinical Analysis, Interlab-UMU, Regional Campus of International Excellence 'Campus Mare Nostrum', University of Murcia, Murcia, Spain; ^4^Department of Animal and Food Science, School of Veterinary Science, Autonomous University of Barcelona, Barcelona, Spain; ^5^Dipartimento di Scienze per la Qualità della Vita, University of Bologna, Rimini, Italy; ^6^Monge & C. S.p.A., Monasterolo di Savigliano, Italy

**Keywords:** canine obesity, fecal microbiota, 16S ribosomal (r)RNA gene, oxidative damage, serum antioxidant capacity, oxidative stress, thyroid homeostasis

## Abstract

**Introduction:**

Obesity is the most common nutritional disease in dogs, and is generally managed by caloric restriction. Gut microbiota alteration could represent a predisposing factor for obesity development, which has been associated with a low-grade inflammatory condition and an impaired antioxidant status. Besides, weight loss has been shown to influence the gut microbiota composition and reduce the inflammatory response and oxidative stress.

**Method:**

However, these insights in canine obesity have not been fully elucidated. The aim of this study was to assess the differences in serum and inflammatory parameters, antioxidant status, fecal microbiota and bacterial metabolites in 16 obese and 15 lean client-owned dogs and how these parameters in obese may be influenced by caloric restriction. First, for 30 days, all dogs received a high-protein, high-fiber diet in amounts to maintain their body weight; later, obese dogs were fed for 180 days the same diet in restricted amounts to promote weight loss.

**Results:**

Before the introduction of the experimental diet (T0), small differences in fecal microbial populations were detected between obese and lean dogs, but bacterial diversity and main bacterial metabolites did not differ. The fecal Dysbiosis Index (DI) was within the reference range (< 0) in most of dogs of both groups. Compared to lean dogs, obese dogs showed higher serum concentrations of acute-phase proteins, total thyroxine (TT4), and antioxidant capacity. Compared to T0, dietary treatment affected the fecal microbiota of obese dogs, decreasing the abundance of Firmicutes and increasing *Bacteroides* spp. However, these changes did not significantly affect the DI. The caloric restriction failed to exert significative changes on a large scale on bacterial populations. Consequently, the DI, bacterial diversity indices and metabolites were unaffected in obese dogs. Caloric restriction was not associated with a reduction of inflammatory markers or an improvement of the antioxidant status, while an increase of TT4 has been observed.

**Discussion:**

In summary, the present results underline that canine obesity is associated with chronic inflammation. This study highlights that changes on fecal microbiota of obese dogs induced by the characteristics of the diet should be differentiated from those that are the consequence of the reduced energy intake.

## 1. Introduction

Canine obesity is a growing global health problem, with data of prevalence ranging between 20% and 40% all over the world (Mao et al., 2013; Montoya-Alonso et al., 2017; Porsani et al., 2020).

Obesity results from a prolonged imbalance of energy intake and expenditure, but it is now understood that the etiology of canine obesity represents a complex interaction of genetics, diet, metabolism, and physical activity levels (Chandler et al., 2017). In addition, the composition and function of the gut microbiota may act as a contributing factor through a variety of proposed mechanisms, including the production of bacterial metabolites that may lead to an increase in dietary energy harvest (Turnbaugh et al., 2006) and promote regulation of adipogenesis and inflammatory adipokines release (Arora et al., 2011). Moreover, in humans, obesity is associated with low-grade systemic inflammation (Das, 2001; Cohen et al., 2021) and weight loss has been shown to reverse this condition (Forsythe et al., 2008), while controversial results have been obtained in dogs (German et al., 2009; Tvarijonaviciute et al., 2012a,c). In addition, recent researches in humans indicate that disturbances in pro-oxidant and anti-oxidant balance play a critical role in the pathogenesis of obesity and may lead to chronic inflammation of the adipose tissue (Marseglia et al., 2015; Sánchez-Rodríguez and Mendoza-Núñez, 2019). To date, the assessment of antioxidant status in canine diseases is still in its early stage (Bastien et al., 2015; Rubio et al., 2016, 2017a; Bosco et al., 2018).

Among the variety of predisposing factors that may have a role in the development of obesity in dogs, owner and lifestyle factors might contribute, since dogs share the same environments with their owners (Lund et al., 2006; Courcier et al., 2010; Muñoz-Prieto et al., 2018; Banton et al., 2022). Studies involving client-owned dogs might be, therefore, more representative of the overall target population (German et al., 2015).

Diet plays a role in regulating the composition and metabolic output of the canine gut microbiota, which adapts to the available nutrients by modulating microbial composition and function (Pilla and Suchodolski, 2021). For that reason, therapeutic approaches throughout gut microbiota regulation in preventing obesity and supporting weight loss in dogs are of interest (Kałużna-Czaplińska et al., 2017; Huang et al., 2020).

In previous studies that have investigated the role of canine obesity and weight loss on gut microbiota, it was challenging to distinguish the effects driven by the experimental diets from those caused by decreased calorie intake, because diet and caloric restriction were usually introduced at the same time (Kieler et al., 2017; Bermudez Sanchez et al., 2020). Caloric restriction represents the main therapeutic strategy for achieving weight loss, and the latter may influence the inflammatory and antioxidant status of obese dogs. However, it remains unclear if the decreased nutrient load consequent to caloric restriction might affect the gut microbiota.

In this study, we first aimed to compare fecal microbiota, fecal bacterial metabolome, and inflammatory and antioxidant status in lean and obese client-owned dogs receiving the same diet with no caloric restriction. Then, we evaluated the changes induced by a weight loss program on these parameters in the obese group.

## 2. Materials and methods

### 2.1. Animals and study design

Sixteen overweight or obese (OB) client-owned dogs (Body Condition Score ≥ 7, according to a 9-point body condition scale chart (Laflamme, 1997)) were prospectively enrolled between July 2019 and September 2020 at the Veterinary Teaching Hospital of the University of Bologna (Italy) for a two-phase study. Additionally, 15 clinically healthy adult private-owned dogs, with a BCS of 4–5/9, were involved in the trial as the lean (CTRL) group for the first phase of the study. To ensure that all dogs were healthy before enrolment, complete patient history was obtained, and a physical examination was performed. Furthermore, all dogs had not received any medicaments, such as antibiotics, that could have an impact on the gut microbiota for at least 90 days before being enrolled in the study. The study protocol was approved by the Scientific Ethics Committee of the University of Bologna.

Body weight (BW) of dogs was measured by electronic weigh scales (KERN & Sohn GmbH 3.0). During the first 30 days (phase 1 of the study), OB and CTRL dogs were fed a dry dog extruded dietetic feed intended for the reduction of excessive body weight (VetSolution Obesity Canine, Monge & C. S.p.a., Monasterolo di Savigliano, Italy, nutrients composition and ingredients are listed in Table 1). During phase 1, the diet was fed in such amounts to maintain the initial body weight of dogs. The maintenance energy requirement (MER) of each dog was calculated using the following equation proposed by FEDIAF (2021) for adult dogs:


$$MER\;\left(\mathrm{kcal}/\mathrm{day}\right)=110\times BW\;{\left(\mathrm{kg}\right)}^{0.75}$$

**Table 1:** Nutrient composition of the experimental diet used in the study (VetSolution Obesity Canine, Monge & C. S.p.a., Monasterolo di Savigliano, Italy).

	AF^a^	DM^b^	Mcal (ME)^c^
Moisture	5.25	/	/
CP	35.0	36.9	109
EE	9.34	9.86	29
Starch	20.4	21.5	63.2
Ash	8.54	9.02	26.5
TDF	17.3	18.3	53.8
Soluble fiber	3.65	3.85	11.3
Insoluble fiber	13.7	14.4	42.5

a Nutrients expressed as g/100 g as fed (AF).

b Nutrients expressed as g/100 g dry matter (DM).

c Nutrients expressed as g/1,000 kcal metabolizable energy (ME) calculated according to NRC-National Research Council (2006).

CP: crude protein; EE: ether extract; TDF: total dietary fiber. Composition: dried chicken meat (20%), tapioca, dried fish (anchovy), pea fiber, potatoes, dried eggs, salmon oil, dried duck meat, brewers’ yeast, plantago seed (1%), minerals, xylooligosaccharides (0.4%), products and by-products from processing fresh fruits and vegetables (melon juice concentrate—Cucumis melo cantalupensis—source of superoxidedismutase (SOD 0.005%), milk protein powder.

where BW is the actual body weight.

For all dogs, the MER was adjusted according to dogs’ habitual energy intake that was estimated based on the information provided by owners. Dogs received only the experimental diet, and any additional foodstuffs (e.g., table scrapes and/or treats) were avoided.

At the end of phase 1 (T30), lean CTRL dogs left the study, while OB dogs moved into phase 2, the weight loss treatment (T30-T210), which lasted 180 days.

During phase 2, OB dogs received the same experimental diet, but a caloric restriction was applied. Individual daily energy amounts were calculated based on the target body weight (TBW) according to the equation proposed by German et al. (2007) that considers both sex and neuter status. The TBW was calculated based on BCS, by estimating that each point above 5 (on the scale of 1 to 9) correlates with about a 10% increase in bodyweight (Laflamme, 2006). The expected rate of weight loss was between 0.5 and 2.0% of starting BW per week (German et al., 2007); the BW and BCS of each dog were assessed every 2 weeks and the dietary plan was adjusted if the weight loss was <0.5% of starting BW per week, reducing the daily ration according to the dog size, by 5 grams (<20 kg BW) or 10 grams (>20 kg BW), as proposed by German et al. (2007).

### 2.2. Sampling collection

During phase 1, fecal samples were collected from each dog at baseline (T0) and after 30 days of dietary treatment (T30). During phase 2, fecal samples from OB dogs were collected after 120 and 210 days of caloric reduction (T120 and T210, respectively). Fecal samples were collected by dog owners after spontaneous defecation and immediately transferred to the laboratory, where they were frozen and kept at −80°C until being processed.

Additionally, a single venous blood sample was collected at T30 from CTRL and OB dogs, and at T120 and T210 from OB animals.

### 2.3. Laboratory analysis

#### 2.3.1. Fecal samples

##### 2.3.1.1. Chemical analysis

The pH was determined after diluting the fecal samples with deionized water at 1:10 (w/v), using a laboratory pH meter (SevenMulti, Mettler Toledo, Greinfesee, Switzerland; accuracy ±0.01), while fecal ammonia was determined using an enzymatic colorimetric test (Urea/BUN-Color; BioSystems S.A., Barcelona, Spain).

The VFA were measured by gas chromatography, according to the method described by Pinna et al. (2021). Biogenic amines were determined by HPLC separation and fluorimetry quantification according to Stefanelli et al. (1986).

##### 2.3.1.2. Real-time quantitative PCR

An aliquot of 100 mg of feces was extracted for DNA with a commercially available kit following manufacturer instructions (PowerSoil® DNA Isolation Kit, MOBIO Laboratories, Inc., Carlsbad, CA, USA). Quantitative PCR was performed using universal bacteria primers for specific bacterial groups: *Blautia* spp., *Clostridium hiranonis*, *Escherichia coli*, *Faecalibacterium* spp., *Fusobacterium* spp., *Streptococcus* spp., and *Turicibacter* spp., according to a previously described method (Garcia-Mazcorro et al., 2012b). Extracted DNA was quantified, and quality checked, with NanoDrop 2000 spectrophotometer (Thermo Scientific, USA).

Results are expressed as the abundance of DNA for each bacterial group, and logarithms of relative DNA copy number were used to calculate the degree of dysbiosis [DI, AlShawaqfeh et al., 2017)] in feces of lean and obese dogs.

##### 2.3.1.3. 16S rRNA gene sequencing

The V4 region of the 16S rRNA gene was sequenced at Mr. Dna Laboratory (Molecular Research LP, Mr. DNA, Shallowater, TX) using primers 515F [5′-GTGYCAGCMGCCGCGGTAA, Parada et al., 2016] to 806RB [5′-GGACTACNVGGGTWTCTAAT, Apprill et al., 2015]. Briefly, amplification was performed under the following conditions: 95°C for 5 min, followed by 30 cycles of 95°C for 30 s, 53°C for 40 s and 72°C for 1 min, after which a final elongation step at 72°C for 10 min. After amplification, PCR products were checked in 2% agarose gel to determine the success of amplification and the relative intensity of bands. Samples were multiplexed using unique dual indices and pooled together in equal proportions based on their molecular weight and DNA concentrations. Pooled samples were purified using calibrated Ampure XP beads. Then the pooled and purified PCR product was used to prepare an Illumina DNA library. Sequencing was performed at on a MiSeq following the manufacturer’s guidelines. Sequences of the 16S rRNA genes were processed using Quantitative Insights Into Microbial Ecology 2 [QIIME 2, v 2018.6, Bolyen et al., 2019]. The raw sequences were uploaded to NCBI Sequence Read Archive under accession number PRJNA822358. The sequence data was demultiplexed, and an amplicon sequence variant (ASV) table was created using DADA2 (Callahan et al., 2016). Prior to downstream analysis, sequences assigned as chloroplast, mitochondria, and low abundance OTUs, containing less than 0.01% of the total reads in the dataset were removed. Samples were rarefied to 4,990 sequences per sample, based on the lowest read depth, to normalize sequencing depth across all samples. Alpha diversity was evaluated with Chao 1, Shannon diversity, and observed species. Beta diversity was evaluated by weighted an unweighted UniFrac distance matrices and visualized using PCoA (Principal Coordinate Analysis) plots.

#### 2.3.2. Blood samples

Blood samples were collected after 15 h of fasting, at T30 in CTRL and OB dogs and then at T120 and T210 in OB dogs, to assess a complete blood cell count (CBC) and serum biochemistry profile. The CBC was performed with an automated hematology analyzer (ADVIA 2120, Siemens Healthcare Diagnostics, Tarrytown NY, USA), while chemistry parameters were carried out on an automated chemistry analyzer (AU480, Beckman Coulter/Olympus, Brea, California, USA). Blood samples for the determination of all the biochemistry variables were collected in serum separating tubes. Coagulated blood samples were centrifuged for 10 min at 3,000 *g*; the serum was immediately transferred to plastic tubes, stored at 4°C and analyzed the same day, or stored at −80°C and thawed immediately before analysis. Blood samples for the determination of CBC and chemistry parameters were analyzed the same day of the sampling in all dogs. Inflammatory markers such as C-reactive protein (CRP) and haptoglobin (Hp) were assayed afterward on stored frozen samples obtained from CTRL dogs at T30 and OB dogs at T30 and T210. The CRP concentration was determined by an immunoturbidimetric assay (Beckman Coulter OSR6147, Beckman Coulter Inc., Brea, California) previously validated in dogs (Gentilini et al., 2005). The Hp concentration was determined using an immunoturbidimetric method that had previously been validated for dogs (Mastrorilli et al., 2007). Total T4 was measured using a chemiluminescent enzyme immunoassay (Immulite 2000, Siemens Healthcare) validated for dogs (Singh et al., 1997).

##### 2.3.2.1. Antioxidant capacity

Analyses were performed on stored frozen serum samples obtained from CTRL dogs at T30 and OB dogs at T30 and T210. Cupric reducing antioxidant capacity (CUPRAC), ferric reducing ability of plasma (FRAP), trolox equivalent antioxidant capacity (TEAC) using acidic medium (TEACA), and the TEAC using the horseradish peroxidase (TEACH) were measured to determine the total antioxidant capacity (TAC) of the samples as previously described in dogs (Rubio et al., 2017b). Total serum thiol was measured according the method described by Jocelyn (1987). The serum enzymes butyrylcholinesterase (BChE) and paraoxonase type 1 (PON1) were analyzed as previously described in serum of dogs (Tvarijonaviciute et al., 2012b). All analyses were performed using the autoanalyzer Olympus AU400 (Olympus Diagnostica GmbH, Ennis, Ireland).

##### 2.3.2.2. Oxidant biomarkers

Analyses were performed on stored frozen serum samples obtained from CTRL dogs at T30 and OB dogs at T30 and T210. Thiobarbituric acid reactive substances (TBARS) were measured as described by Buege and Aust (1978) by using a microplate reader (Powerwave XS, Biotek instruments, Carson City, NV). Reactive oxygen species (ROS) levels were assessed by luminol-mediated chemiluminescence assay (Vong et al., 2014). The resulting chemiluminescence was measured using a microplate reader (Victor 21,420 Multilabel Counter; PerkinElmer, Finland) and results were expressed in counts per second (cps). Ferrous oxidation-xylenol orange (FOX) was measured according to the colorimetric method described by Arab and Steghens (2004) and was performed using the Olympus AU400 Automatic Chemistry Analyser.

### 2.4. Statistical analysis

The D’Agostino and Pearson omnibus normality test was used to assess the normality of the data with parametric distribution. All values are presented in the text as the group mean ± SD for normally distributed data and the median (range) if they were not normally distributed.

A 2-way ANOVA was applied to determine the significance of the changes in fecal pH, fecal chemical parameters, microbial communities, and alpha-diversity indices, both temporal within-group (effect induced by dietary treatment during phase 1 of the study) and between-groups (OB vs. CTRL). If indicated, data were logarithmic transformed before statistical analysis with ANOVA.

To evaluate the influence of caloric restriction (phase 2) on fecal chemical parameters, microbial communities, and alpha-diversity indices among the sample collection time points, a repeated measures ANOVA or the non-parametric analog (Friedman test) were used, following the distribution of data. For all multiple testing, *p*-value was adjusted to *q-*value using Benjamini-Hochberg false-discovery rate correction (FDR) and significance was set at *q* < 0.05.

Multivariate analysis was performed on the unweighted UniFrac distance matrixes using ANOSIM (Analysis of Similarity) test within PRIMER 7 software (PRIMER-E Ltd., Luton, UK) to analyze differences in microbial communities.

To determine differences in blood parameters between OB and CTRL, and before and after caloric restriction in OB dogs, unpaired t-test or the Mann–Whitney test, as well as paired t-test or Wilcoxon matched-pairs were used as appropriate. Pearson correlation coefficient was used to assess the relationship between antioxidant capacity and inflammatory markers.

For all statistical analyses, significance was set at *p* < 0.05.

Statistical analyses were conducted using GraphPad Prism version 9.2 (GraphPad Software, San Diego, CA, USA), except for the ANOSIM test that was performed with PRIMER 6 software package (PRIMER-E Ltd., Luton, UK).

## 3. Results

### 3.1. Phase 1—Fecal metabolites and microbiota, and serum analytes in CTRL and OB dogs

#### 3.1.1. Animals

In total, 16 private-owned adult OB dogs and 15 private-owned adult healthy lean dogs were applied. Full details of the baseline characteristics (T0) of CTRL and OB dogs, are given in Supplementary Table 1. No statistical differences were detected between CTRL and OB dogs regarding baseline characteristics (signalments) and body weight. The median age of OB dogs was 66 months (20 to 111 months); 10 dogs were male (5 were neutered and 5 were intact) and 6 were female (4 were neutered and 2 were intact). The median age of CTRL dogs was 72 months (14 to 116 months); 10 dogs were male (6 were neutered and 4 were intact) and 5 were neutered females. All dogs were of different breed and size. At T0, median body weight (BW) of OB dogs was 27.5 kg (4.73 to 64.10 kg) and BCS was 8 (7 to 9), while median BW of CTRL dogs was 24.7 kg (7.85 to 45.8 kg) and BCS was 5 (4 to 5). Median energy allocation, expressed as kcal of ME per kg^0.75^ TBW, was 103 (87 to 186) and 99 (70 to 187) for OB and CTRL dogs, respectively.

#### 3.1.2. Fecal metabolites and microbiota

Both CTRL and OB groups did not show any significant changes in fecal pH, ammonia and VFA (Table 2) in response to a short-term (30 days) dietary treatment with a diet intended for weight loss, given at a daily amount calculated to provide the energy to maintain their body weight, to avoid any potential effect deriving from caloric restriction. Among biogenic amines (Table 2), spermine fecal concentration was markedly decreased by diet within the CTRL group (T30: 104 nmol/g vs. T0: 297 nmol/g, *q* < 0.001). Moreover, compared with CTRL, fecal spermine was higher in OB dogs (*p* = 0.005) both before (OB T0: 360 nmol/g vs. CTRL T0: 297 nmol/g, *q* < 0.001) and after dietary treatment (OB T30: 251 nmol/g vs. CTRL T30: 104 nmol/g, *q* = 0.002); within OB, instead, the fecal concentrations of biogenic amines were not modified by the dietary treatment (Table 2).

**Table 2:** Fecal pH values and concentrations of ammonia (μmol/g), volatile fatty acids (μmol/g) and biogenic amines (nmol/g) in feces of obese (*n* = 16) and lean dogs (*n* = 15) fed the same experimental diet, without caloric reduction, for 30 days (phase 1 of the study).

	OB		CTRL			ANOVA *p-*value		
	T0	T30	T0	T30	SEM	Group	Diet	Group × Diet
pH	6.82	6.79	6.89	6.77	0.044	0.794	0.425	0.618
Ammonia	29.8	33.5	35.0	38.0	1.333	0.060	0.134	0.977
Acetic a.	55.7	61.3	66.4	73.5	2.543	0.024	0.205	0.888
Propionic a.	30.9	27.2	40.3	36.0	1.905	0.017	0.280	0.935
Isobutyric a.	1.73	1.80	1.71	1.72	0.119	0.660	0.577	0.654
*n-*Butyric a.	10.4	10.4	11.9	13.3	0.512	0.030	0.490	0.512
Isovaleric a.	2.54	2.37	2.27	1.99	0.166	0.610	0.714	0.692
Total VFA	102	103	123	126	4.323	0.011	0.742	0.908
Putrescine	1,078	1,010	798	1,087	123	0.514	0.107	0.113
Cadaverine	834	785	443	602	201	0.147	0.010	0.746
Spermidine	560	432	456	385	47.21	0.177	0.859	0.668
Spermine	360^a^	251^a^	297^a^	104^b^	39.24	0.005	<0.001	0.245

OB: obese dogs; CTRL: lean dogs; SEM: standard error of the mean; VFA: volatile fatty acids. Means with different superscripts are significantly different in multiple comparisons with FDR adjustment.

The Shannon, Chao1 and observed operational taxonomic units (OTUs) indices were used to quantify fecal microbiota alpha diversity evenness and richness between OB and CTRL dogs in response to the dietary treatment, without detecting significant differences (*p* > 0.05, Table 3).

**Table 3:** Alpha-diversity indices of fecal microbiota of obese (*n* = 16) and lean dogs (*n* = 15) fed the same experimental diet, without caloric reduction, for 30 days (phase 1 of the study).

	OB		CTRL			ANOVA *p*-value		
	T0	T30	T0	T30	SEM	Group	Diet	Group X diet
No. of observed OTUs	134	159	142	148	7.161	0.888	0.129	0.358
Chao1	136	162	143	150	7.439	0.831	0.126	0.376
Shannon Index	6.45	6.77	6.62	6.65	0.085	0.826	0.140	0.238

OB: obese dogs; CTRL: lean dogs; OTUs: operational taxonomic units; SEM: standard error of the mean.

**Figure 1:**
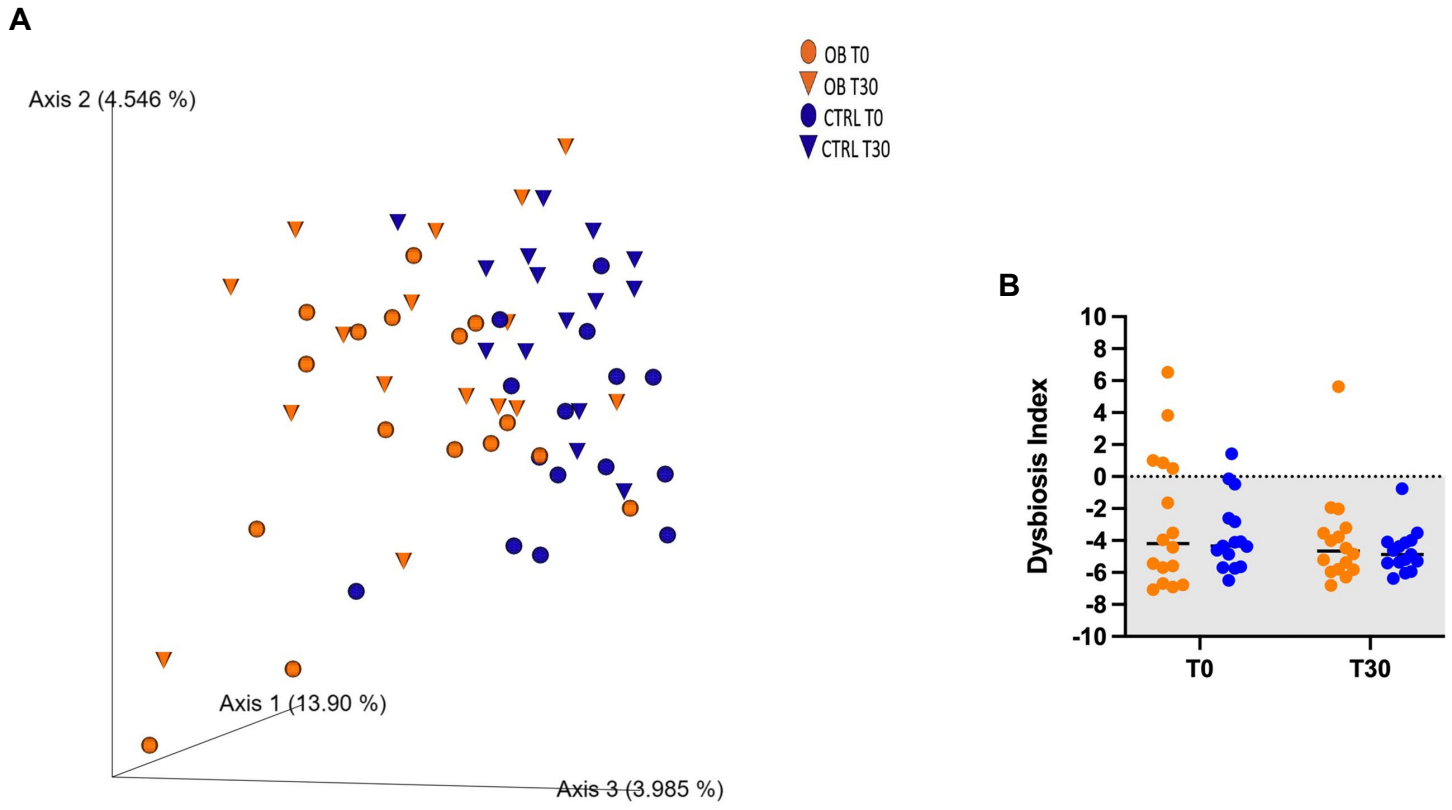
**(A)** PCoA plot based on the unweighted UniFrac distance metric of the fecal microbiota of obese (OB, orange, dot = T0; triangle = T30) and lean dogs (CTRL, blue, dot = T0, triangle = T30; **B**) qPCR-based fecal Dysbiosis Index of obese and lean dogs at trial start (T0) and after (T30) dietary treatment. Negative values (the grey area) are indicative of a healthy microbiota, while values between 0 and 2 are considered equivocal.

Unweighted UniFrac analysis of similarities showed that there not were differences between OB and CTRL dogs at T0 (OB vs. CTRL, *p* = 0.070 and *R* = 0.076, Figure 1), nor after dietary treatment (T30: OB vs. CTRL, *p* = 0.166 and *R* = 0.028, Figure 1A). The dietary treatment, in fact, failed to affect the β-diversity within microbial communities of each group (OB T0 vs. T30, *p* = 0.108 and *R* = 0.045; CTRL T0 vs. T30, *p* = 0.062 and *R* = 0.084, Figure 1A). In the same way, the DI (Figure 1B) did not differ between groups. In OB dogs, in fact, the bacterial abundances were mostly within the reference intervals established for healthy dogs (Supplementary Table 2).

**Figure 2:**
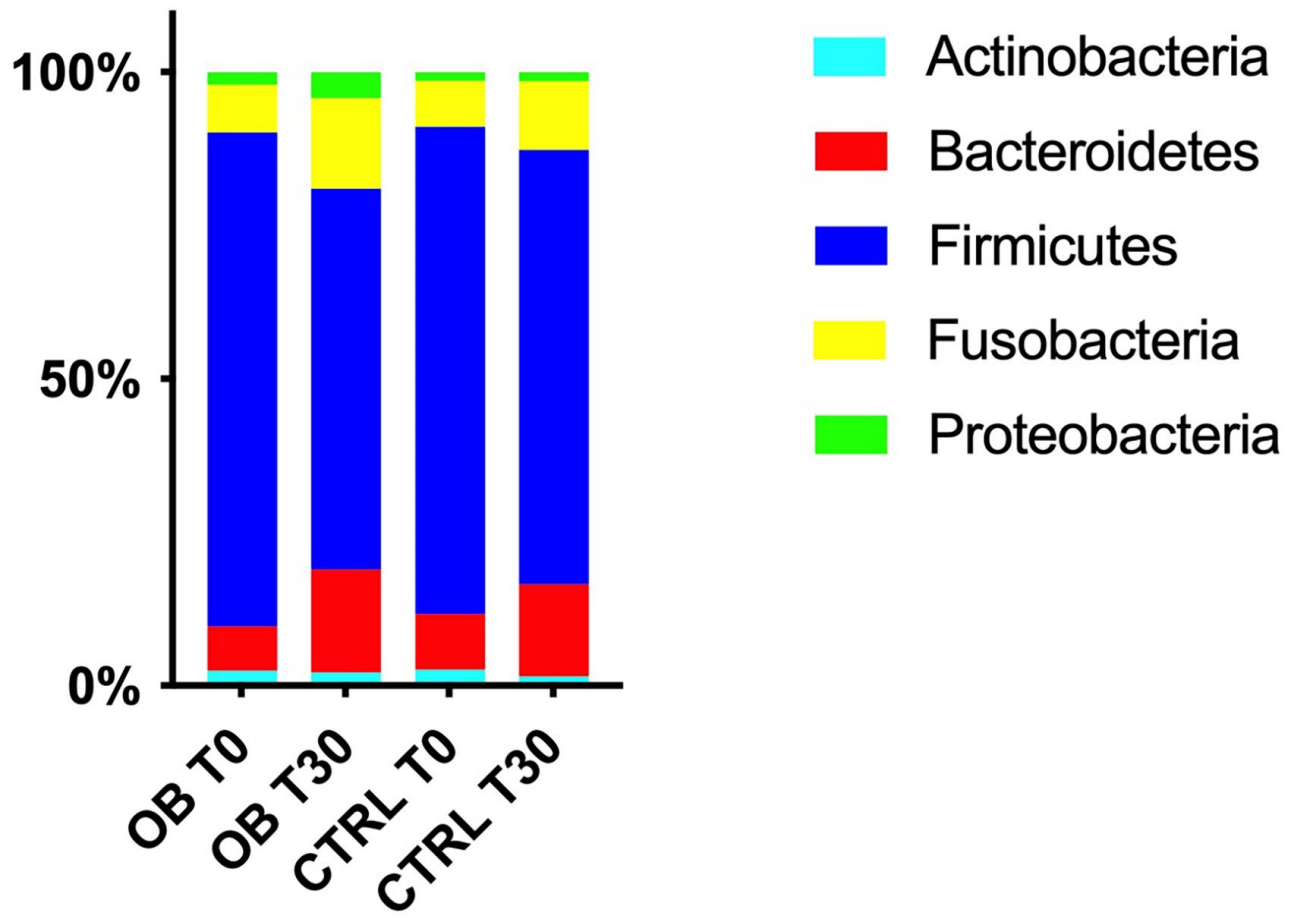
Relative abundances of the 5 most abundant phyla in fecal samples of obese and lean dogs at trial start (T0) and after (T30) dietary treatment. In obese dogs, the median abundance of Firmicutes decreased after dietary treatment (T0: 86% vs. T30: 59%, *q* = 0.010), while a tendency to an increase was observed for Bacteroidetes and Fusobacteria (T0: 4.5% vs. T30: 19%, *q* = 0.065 and T0: 4.4% vs. T30: 15%, *q* = 0.054 for Bacteroidetes and Fusobacteria, respectively).

**Figure 3:**
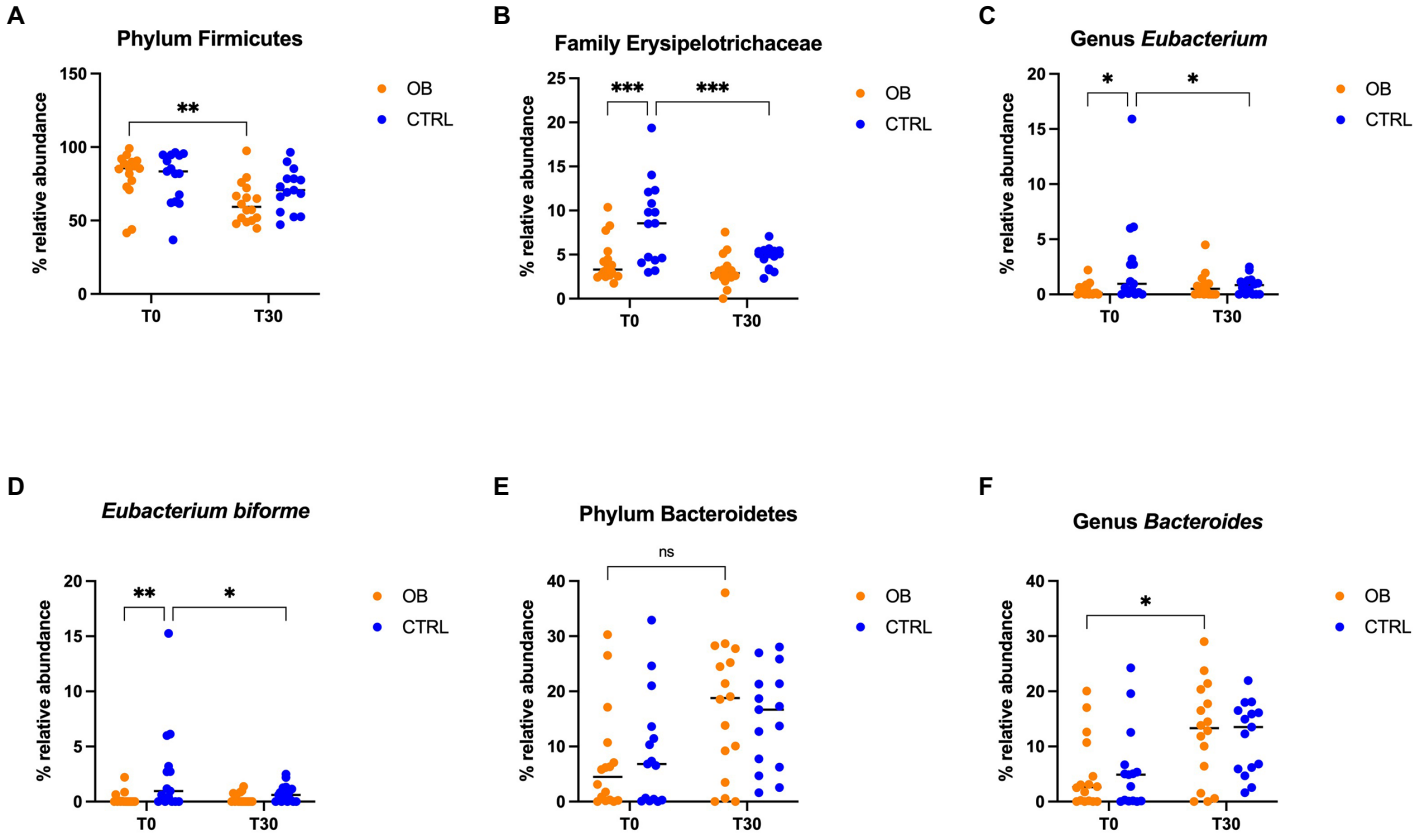
Relative abundance of selective bacterial populations: **(A)** phylum Firmicutes, **(B)** family Erysipelotrichaceae, **(C)** genus *Eubacterium*, **(D)** species *Eubacterium biforme*, **(E)** phylum Bacteroidetes, **(F)** genus Bacteroides, in fecal samples of obese and lean dogs before (T0) and after (T30) dietary treatment. Significance level: *q < 0.05, **q < 0.01, ***q < 0.001, ns indicate *p*-values that did not pass FDR correction (*q* = 0.08, *p* < 0.05).

At phyla level, five different phyla were identified as the most abundant in OB and CTRL fecal samples, with no differences between groups (*q* > 0.05, Figure 2). Firmicutes were predominant in OB and CTRL both before and after dietary treatment (range 41–99% and 37–96% for OB and CTRL, respectively), followed by Bacteroidetes (range 0–38% and 0–33% for OB and CTRL, respectively) and Fusobacteria (range 0–29% for both OB and CTRL). In OB dogs, the median abundance of Firmicutes decreased at T30 (T0: 86% vs. T30: 59%, *q* = 0.010, Figure 3A), while dietary treatment did not affect the bacterial populations of healthy dogs.

Differences in terms of bacterial phylum, class, order, family, genera and species between OB and CTRL dogs are available in Supplementary File 1, while significative results are presented in Figure 3; within the phylum Firmicutes, relative abundance of the family Erysipelotrichaceae differed between OB and CTRL (Figure 3B) before dietary treatment started (OB: 3.31% vs. CTRL: 8.56%, *q* < 0.001); moreover, abundance of Erysipelotrichaceae in CTRL dogs was reduced by dietary treatment (T0: 8.56% vs. T30: 5.10%, *q* = 0.001). The same was observed for the genus *Eubacterium* (Figure 3C: OB: 0.12% vs. CTRL: 0.96%, *q* = 0.039 and CTRL T0: 0.96% vs. T30: 0.84%, *q* = 0.048), with *E. biforme* being the most represented species of this genus (Figure 3D: OB T0: 0% vs. CTRL T0: 0.96%, *q* = 0.008 and CTRL T0: 0.96% vs. T30: 0.60%, *q* = 0.034). Abundance of the genus *Bacteroides* (family Bacteroidaceae, phylum Bacteroidetes, Figure 3F) increased in OB in response to the dietary treatment (T0: 2.60% vs. T30: 13.3%, *q* = 0.029, Figure 3F).

#### 3.1.3. Serum analytes

Among serum biochemical parameters collected in both groups at T30 (Table 4; Supplementary Table 3), total protein (*p* = 0.033), C-reactive protein (CRP, *p* = 0.007), Hp (*p* = 0.003), Trolox equivalent antioxidant capacity (TEACH, *p* = 0.037), and Thiol (*p* = 0.013) were higher in OB than in CTRL dogs, while the opposite was true for phosphate (*p* = 0.008). However, all phosphate values were within the normal range. Moreover, OB tended to have higher total thyroxine (TT4) than CTRL (*p* = 0.07).

**Table 4:** Serum concentrations of acute phase proteins (CRP and Hp), inflammatory and oxidative stress biomarkers (median [range]) in obese (*n* = 16) and lean (*n* = 15) dogs fed the same experimental diet, without caloric restriction, for 30 days (phase 1 of the study), and in the same obese dogs after 180 days of caloric restriction (phase 2 of the study).

Parameter	CTRL T30	OB T30	OB T210
CRP (mg/dl)	0.91 [0.80–2.4]	1.1 [0.8–2.7]	0.96 [0.74–4.5]
RI [0–0.85]			
*p*-value	**0.007**		0.242
Hp (mg/dl)	30 [1–125]	85 [16–197]	74 [3–157]
RI [20–140]			
*p*-value	**0.003**		0.395
PON1 (IU/L)	3.63 [2.62–4.08]	3.66 [2.40–4.40]	3.66 [2.74–4.60]
*p*-value	0.310		0.941
CUPRAC (mmol/L)	0.44 [0.37–0.63]	0.48 [0.36–0.62]	0.52 [0.34–0.61]
*p*-value	0.079		0.785
FRAP (mmol/L)	0.44 [0.30–0.63]	0.48 [0.31–0.61]	0.49 [0.33–0.64]
*p*-value	0.271		0.972
TEACH (mmol/L)	0.65 [0.57–0.85]	0.73 [0.54–0.81]	0.71 [0.54–0.82]
*p*-value	**0.037**		0.374
TEACA (mmol/L)	0.29 [0.22–0.43]	0.29 [0.22–0.37]	0.29 [0.22–0.38]
*p*-value	0.955		0.531
Thiol (μmol/L)	180 [108–273]	251 [110–384]	215 [93–366]
*p*-value	**0.013**		0.343
BChE (U/mL)	5.00 [3.30–8.40]	5.10 [3.60–8.50]	5.55 [3.80–8.30]
*p*-value	0.942		0.626
TBARS (μmol/L)	1.83 [1.2–3.1]	2.19 [0.92–3.24]	2.79 [1.13–4.30]
*p*-value	0.126		0.201
FOX (μmol/L)	52.04 [22.68–101]	51.96 [23.9–89.1]	55.58 [23.7–74.5]
*p*-value	0.646		0.506
ROS (cps)	1,510 [828–2,368]	1,476 [1108–2052]	1,630 [994–2,314]
*p*-value	0.481		0.133

OB: obese dogs; CTRL: lean dogs. BChE: butyrylcholinesterase; CRP: c-reactive protein; CUPRAC: cupric reducing antioxidant capacity; FOX: ferrous oxidation-xylenol orange; FRAP: ferric reducing ability; Hp: haptoglobin; PON1: paraoxonase 1; ROS: reactive oxygen species; TBARS: thiobarbituric acid reactive substances; TEACA, TEACH: trolox equivalent antioxidant capacity. *p*-value quoted are for parametric or not parametric tests comparing CTRL and OB T210 with OB T30. Data in bold highlight statistical significance.

The Pearson correlation test revealed that, in OB dogs, serum Hp concentrations negatively correlated with the following antioxidant capacity biomarkers (Table 5): TEACH (*r* = −0.55; *p* = 0.029), TEACA (*r* = −0.59; *p* = 0.017), Thiol (*r* = −0.52; *p* = 0.039). There was instead no correlation between CRP and antioxidant capacity biomarkers in OB.

**Table 5:** Coefficients of correlation between serum biomarkers of antioxidant capacity (TEACH, TEACA and Thiol) and serum inflammatory markers (CRP and Hp) in obese dogs (*n* = 16) before (T30) and after (T210) caloric restriction (phase 2 of the study).

	CRP (mg/dl)		Hp (mg/dl)	
	*r*	*p*	*r*	*p*
TEACH (mmol/L) T30	−0.119	0.661	−0.546	**0.029**
T210	0.144	0.595	0.024	0.930
TEACA (mmol/L) T30	0.069	0.798	−0.588	**0.017**
T210	0.153	0.570	0.278	0.297
Thiol (μmol/L) T30	−0.460	0.073	−0.520	**0.039**
T210	−0.252	0.346	−0.311	0.242

CRP: c-reactive protein; Hp: haptoglobin; TEACA, TEACH: trolox equivalent antioxidant capacity. Data in bold highlight statistical significance.

### 3.2. Phase 2—Fecal metabolites and microbiota, and serum analytes in OB dogs

#### 3.2.1. Weight loss outcomes

Full details of weight loss outcomes of OB dogs are given in Table 6. All dogs lost weight, and 3 out of 16 reached their target weight. The median overall percentage of weight loss was 12.9% (7.3% to 22.3%) of starting body weight (SBW) and, in detail, BW changed by a median of-9% from T30 to T120 (−3.5% to −12.4%) and −6% from T120 to T210 (+0.6% to −11.4%). The median overall rate of weight loss, expressed as a percentage of SBW lost per week, was 0.53% (0.3% to 0.9%), and the rate of weight loss changed by a median of 0.74% from T30 to T120 (0.3% to 1%), to a median of 0.49% from T120 to T210 (−0.06% to 1%). The median overall energy intake, expressed as kcal of ME per kg^0.75^ TBW, during caloric restriction was 69 (55 to 101).

**Table 6:** Weight loss outcomes of obese dogs (*n* = 16) before (T30), after 3 months (T120), and at the end (T210) of caloric restriction (phase 2 of the study).

Parameter	T30	T120	T210	Overall
Body weight (kg)	27.5 [4.6–63.7]	25.4 [4.10–58]	23.4 [3.6–58.7]	
BCS^a^	8 [7–9]	7 [6–9]	7 [4–8]	
Weight loss (%)^b^		9.0 [3.5–12.4]	6.0 [−0.6–11.4]	12.9 [7.3–22.3]
Target body weight reached, *n*. OB (%OB)		3 (19%)		
Rate of weight loss^c^		0.74 [0.3–1.0]	0.49 [−0.06–1.0]	0.53 [0.3–0.9]
Daily energy intake^d^	72 [64–105]	69 [57–102]	64 [41–95]	69 [55–101]

All data, except for the number of dogs that reached the target body weight, are expressed as median [range].

a BCS based on 9 point scale (Laflamme, 1997).

b Expressed as the percentage of starting body weight. Positive values indicate a net loss, negative value indicate a net gain.

c Expressed as the percentage of starting body weight lost per week.

d Expressed as kcal of ME per kg^0.75^ of target body weight.

#### 3.2.2. Fecal metabolites and microbiota

Fecal pH, ammonia, VFA, and biogenic amines were not affected by caloric restriction (*p* > 0.05, Table 7).

**Table 7:** Fecal pH values and concentrations of ammonia (μmol/g), volatile fatty acids (μmol/g) and biogenic amines (nmol/g) in feces of obese dogs (*n* = 16) before (T30), after 90 days (T120) and at the end (T210) of caloric restriction (phase 2 of the study).

	T30	T120	T210	Pooled SEM	*p*-value
pH	6.79	6.78	6.79	0.067	0.988
Ammonia	33.5	37.0	30.8	2.406	0.135
Acetic a.	61.3	56.9	60.8	3.503	0.624
Propionic a.	27.2	24.7	34.1	2.472	0.031
Isobutyric a.	1.80	1.47	1.76	0.165	0.509
*n-*Butyric a.	10.4	10.3	11.4	0.719	0.504
Isovaleric a.	2.37	2.08	2.48	0.263	0.820
Total VFA	104	95.5	111	5.568	0.176
Putrescine	1,010	1,134	1,163	123	0.306
Cadaverine	785	598	551	99.6	0.378
Spermidine	432	452	456	40.7	0.804
Spermine	251	237	269	32.7	0.965

SEM: standard error of the mean; VFA: volatile fatty acids.

**Figure 4:**
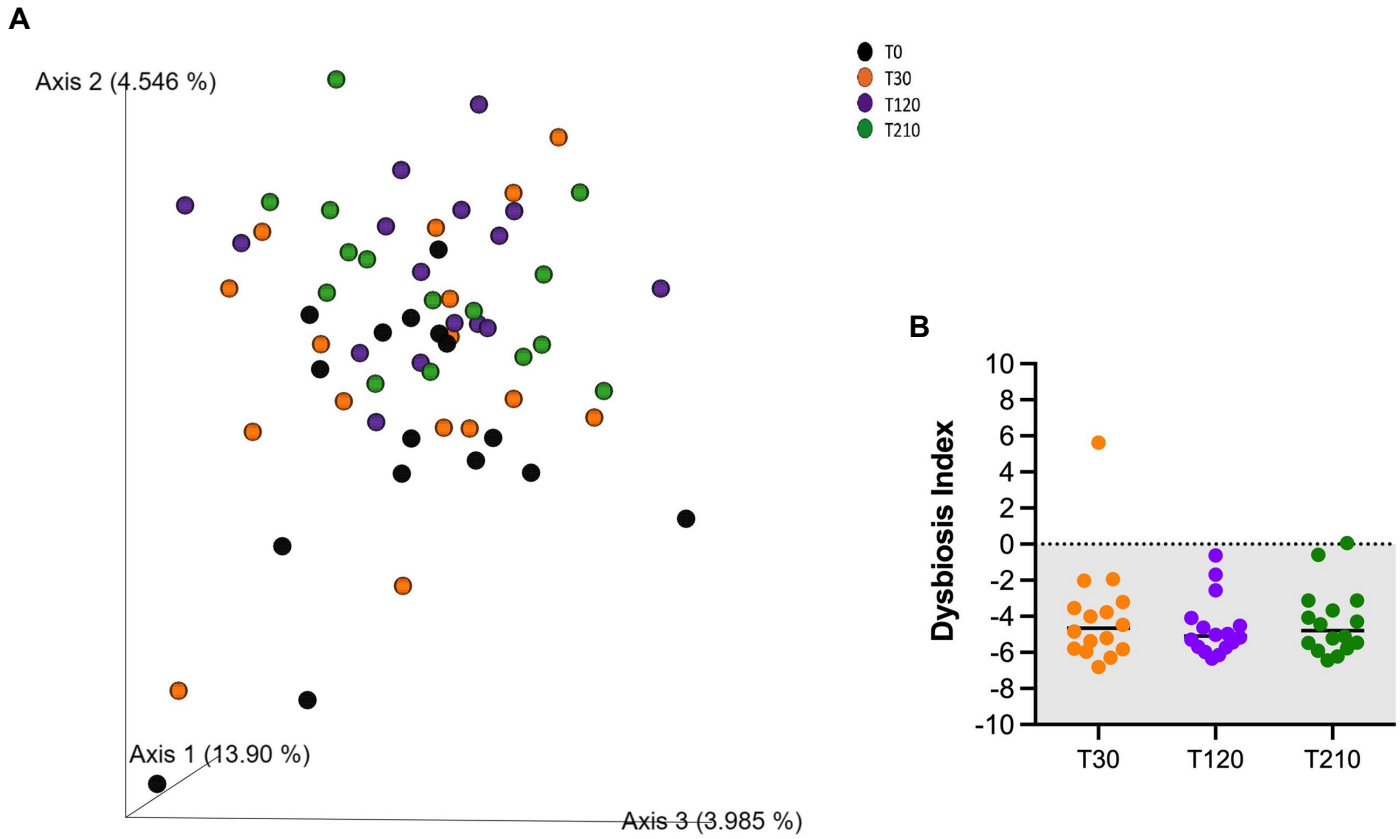
**(A)** PCoA plot based on the unweighted UniFrac distance metric of the fecal microbiota of obese dogs. Differences before (T30) and after 90 and 180 days of caloric restriction were not observed (*p* > 0.05). **(B)** qPCR-based fecal Dysbiosis Index of obese dogs before (T30), after 90 days (T120) and at the end (T210) of caloric restriction. Negative values (the grey area) are indicative of a health microbiota, while values between 0 and 2 are considered equivocal.

Similarly, alpha-and beta-diversity indices, as well as selected bacterial populations detected by qPCR were not influenced by caloric restriction (Table 8; Figure 4A; Supplementary Table 4, respectively). Also, DI was not affected by caloric restriction in OB dogs (Figure 4B).

**Table 8:** Alpha-diversity indices of fecal microbiota of obese dogs (*n* = 16) before (T30), after 90 days (T120), and at the end (T210) of caloric restriction (phase 2 of the study).

	T30	T120	T210	Pooled SEM	*p*-value
No. of observed OTUs	159	155	150	7.556	0.719
Chao1	162	157	155	7.807	0.811
Shannon Index	6.77	6.71	6.68	0.081	0.739

OTUs: operational taxonomic units; SEM: standard error of the mean.

**Figure 5:**
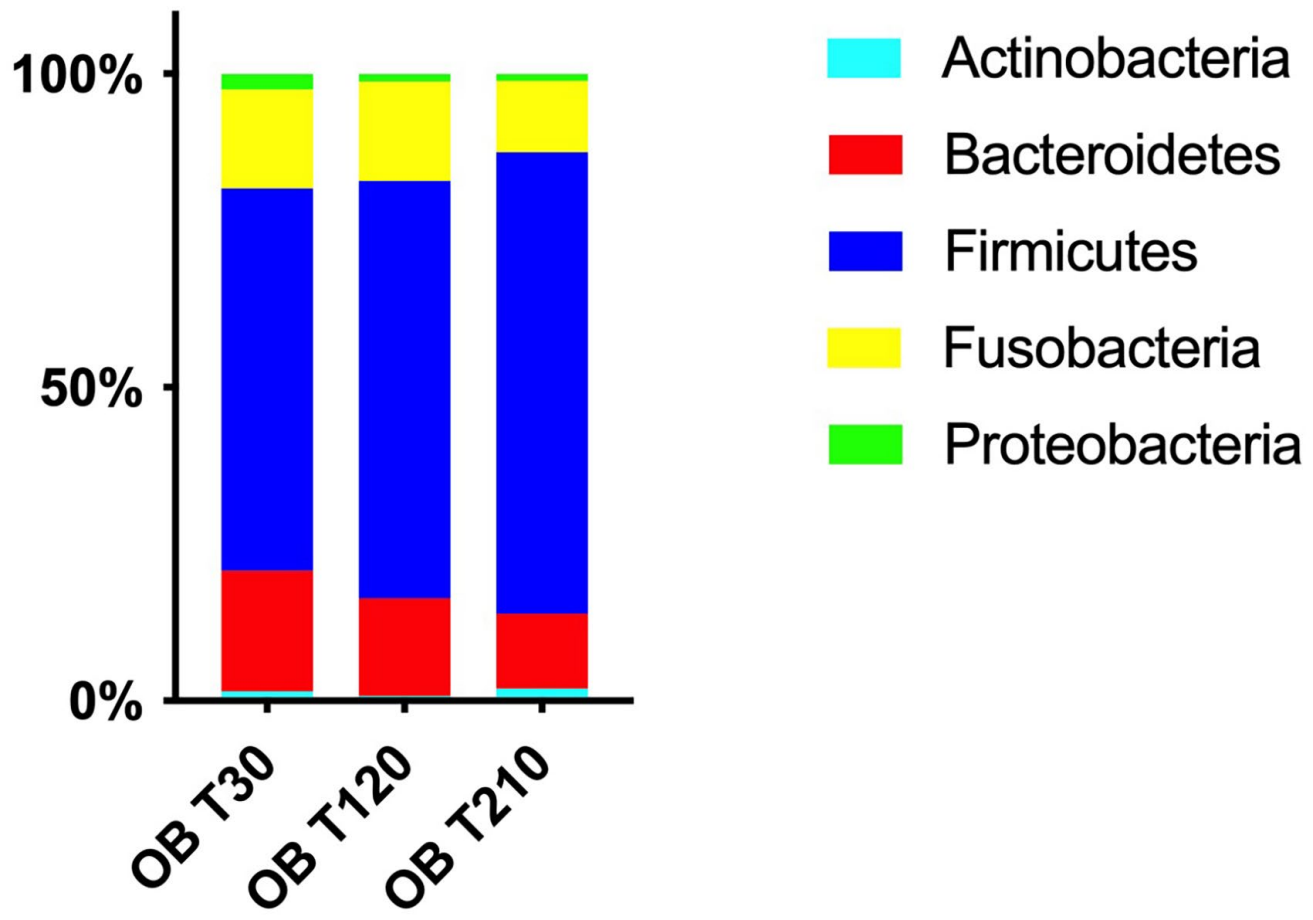
Relative abundances of the 5 most abundant phyla in fecal samples of obese dogs before (T30), after 90 days (T120) and at the end (T210) of caloric restriction; the median abundance of Actinobacteria numerically decrease at T120 compared with T30 (*q* > 0.05) and then significantly increased at T210 compared with T120 (1.9% vs. 0.7%, *q* = 0.014). Moreover, a tendency to a decrease was observed between T30 vs. T120 and T120 vs. T210 for Bacteroidetes (T30: 19% vs. T120: 14% and T120: 14% vs. T210: 13%, *q* = 0.051) and between T30 vs. T120 for Proteobacteria (T30: 2.4% vs. T120: 1.1%, *q* = 0.051).

Among the five most abundant bacterial phyla recovered in OB dogs in Phase 1, only Actinobacteria showed a shift in their community composition during caloric restriction (Figure 5): in particular, the median abundance of Actinobacteria was lower at T120 than as at T30 (0.7% vs. 1.4%, range: 0–2.1 vs. 0–7.2, *q* > 0.05) and then significantly increased at T210 compared with T120 (median: 1.9% vs. 0.7% range: 0.3–6.2 vs. 0–2.1, *q* = 0.014).

**Figure 6:**
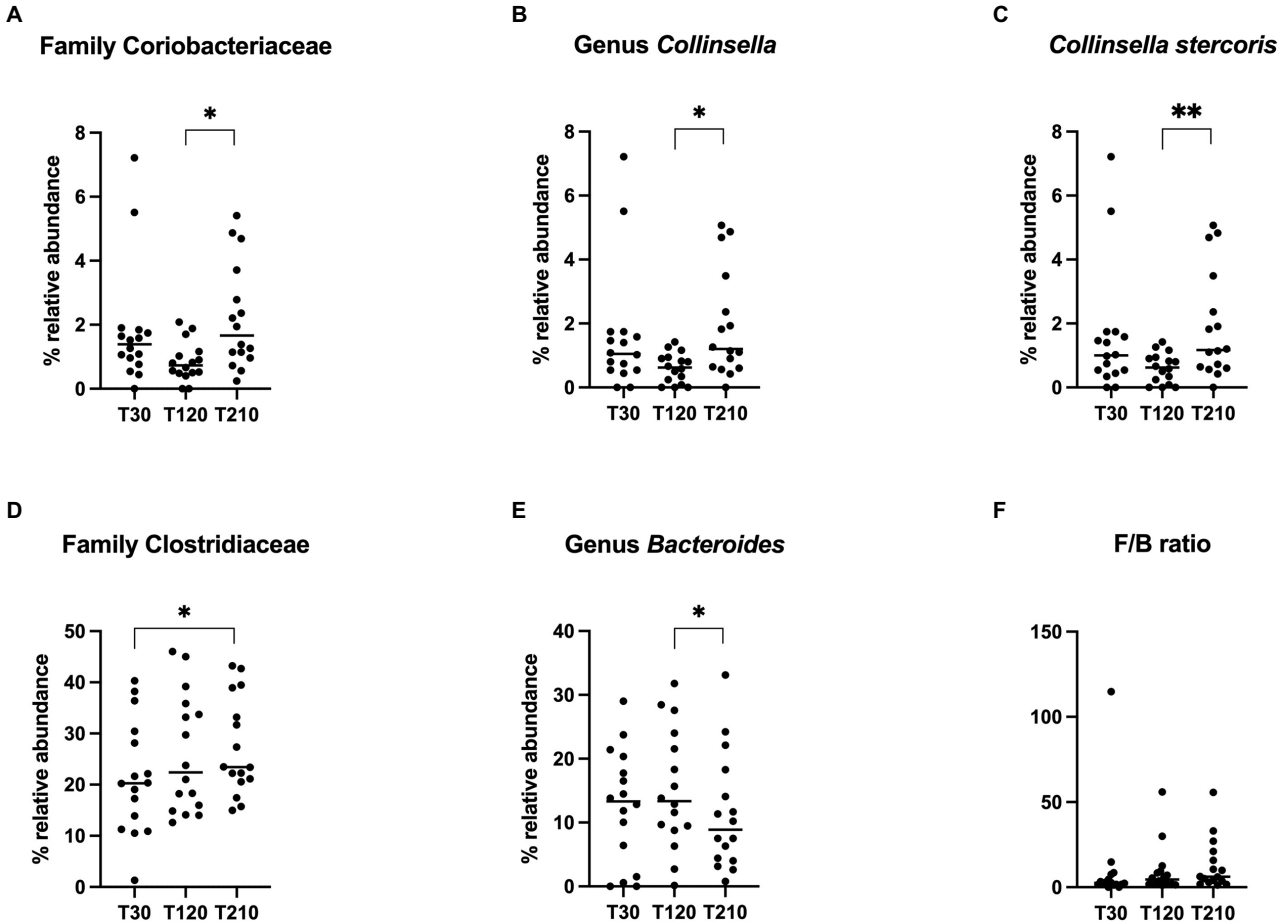
Relative abundance of selective bacterial populations: **(A)** family Coriobacteriaceae, **(B)** genus *Collinsella,* **(C)** species *Collinsella stercoris*, **(D)** family Clostridiaceae, **(E)** genus *Bacteroides,* **(F)** Firmicutes/Bacteroidetes ratio, in fecal samples of obese dogs before (T30), after 90 days (T120) and at the end (T210) of caloric restriction. Significance level: *q < 0.05, **q < 0.01.

Differences in terms of bacterial class, order, family, genera and species in OB dogs during caloric restriction, are available in Supplementary File 1, while significative results are presented in Figure 6. At the family level (Figure 6A), fecal microbiota composition of OB dogs was characterized by an increase of the family Coriobacteriaceae after the second half of the caloric restriction period (T120: 0.73% vs. T210: 1.66%, *q* = 0.018), even if their bacterial abundance at T210 was not different from the one observed at T30 (T30: 1.39% vs. T210: 1.66%, *p* > 0.05). The same trend was observed at genus and species levels for the genus *Collinsella* (T120: 0.62% vs. T210: 1.20%, *q* = 0.014, Figure 6B) and in particular for *C. stercoris* (T120: 0.62% vs. T210: 1.17%, *q* = 0.008, Figure 6C). The relative abundance of the family Clostridiaceae increased significantly after caloric restriction (T210: 23% vs. T30: 20%, *q* = 0.04, Figure 6D), while the abundance of the genus *Bacteroides* instead decreased after the second half of the weight loss period (T120: 13% vs. T210: 8.9%, *q* = 0.032, Figure 6E).

#### 3.2.3. Serum analytes

**Figure 7:**
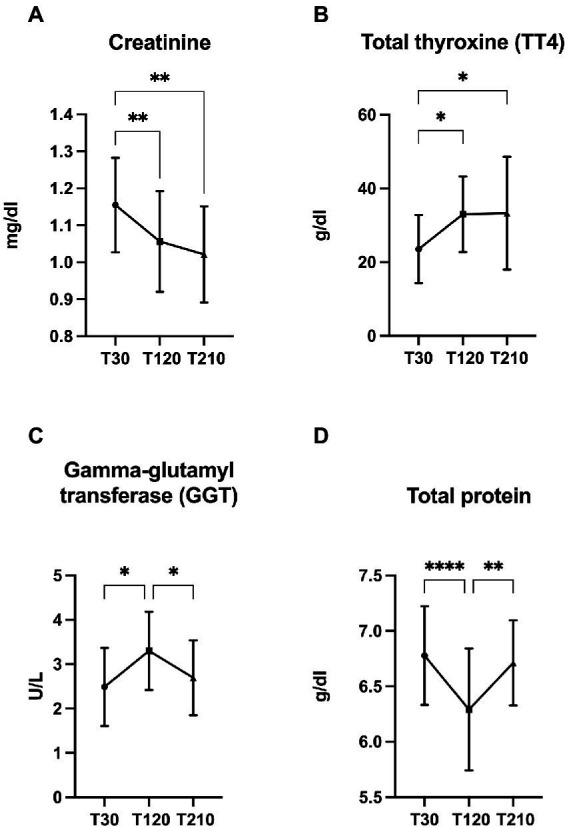
Significant changes in the concentration of selected serum metabolites. **(A)** creatinine, **(B)** total protein, **(C)** gamma-glutamyl transferase (GGT), and **(D)** total thyroxine (TT4) in obese dogs (*n* = 16) before (T30), after 90 days (T120), and at the end (T210) of caloric restriction. Significance level: *q < 0.5, **q < 0.01, ****q < 0.0001.

All serum biochemical parameters are presented in Supplementary Table 5, and significant changes found in OB dogs during caloric reduction are showed in Figure 7. Compared with T30, a decrease of creatinine (*p* < 0.0001, Figure 7A) and an increase of TT4 serum concentrations (*p* = 0.007, Figure 7B) were observed at T120 and T210. At T120 serum total protein and gamma-glutamyl transferase (GGT) concentrations decreased (*p* < 0.0001, Figure 7C) and increased (*p* = 0.007, Figure 7D), respectively, while at T210 it returned to values similar to those observed at T30.

Caloric restriction did not result in any changes in inflammatory and oxidative status biomarkers (Table 4); moreover, there was no correlation between biomarkers of antioxidant capacity (TEACH, TEACA and Thiol) and inflammatory markers (CRP and Hp; Table 5).

## 4. Discussion

Obesity is the most commonly occurring metabolic disease in dogs, causing severe concurrent clinical conditions, such as functional (Mosing et al., 2013; Tropf et al., 2017) and metabolic disorders (Tvarijonaviciute et al., 2012c), and thus decreasing quality of life and longevity (Kealy et al., 2002; Courcier et al., 2010; German et al., 2012).

When it comes to an excess of body fat in dogs, the protracted imbalance between food intake and energy expenditure must be considered (Courcier et al., 2010), as well as factors related to changes in eating behavior and voluntary physical activity, such as neutering and ageing (Courcier et al., 2010; Kawauchi et al., 2017; Suarez et al., 2022). Such factors might have affected energy expenditure in client-owned OB dogs in this study, which had different age and neuter status. The energy intake assessed in OB dogs, before applying the caloric restriction, showed values similar to what has been considered as appropriate for adult dogs with 1–3 h/day activity level (FEDIAF, 2021). However, the daily activity level of OB dogs was not assessed in the present study.

### 4.1. Thyroid function and serum biochemical profile

Excess body fat is also related to endocrine and inflammatory profile changes in dogs with insulin resistance, modification of adipokine patterns, and lipid metabolism changes being reported (Antonio Brunetto et al., 2011; Clark and Hoenig, 2016). In this study, we observed significant differences in TT4 and acute phase proteins (CRP and Hp) concentrations between a population of 16 client-owned obese and 15 lean dogs.

Thyroid hormones are among the main factors involved in the regulation of energy expenditure (Reinehr, 2010; Mullur et al., 2014) and thyroid dysfunctions are responsible of significant changes in body weight in dogs, with hypothyroidism being usually associated with weight gain, reduced thermogenesis and metabolic rate (Scott-Moncrieff, 2015a,b). Although thyroid function is usually normal in obese dogs, they are frequently tested for hypothyroidism before starting a weight-loss protocol. Differences in thyroid function have been previously evaluated in obese and lean dogs showing that obese dogs have higher total triiodothyronine (TT3) and TT4 serum concentrations than lean animals, even if without clinical importance (Daminet et al., 2003). In accordance with these findings, in the present study, TT4 concentrations of OB dogs before caloric restriction tended to be higher than in CTRL animals, but remained within the normality range. During two previous studies (Daminet et al., 2003; Diez et al., 2004), a decrease of free thyroxine (fT4) and TT3 was observed in Beagle dogs following a weight loss program, leading authors to suppose that thyroid homeostasis of dogs may be affected by energy restriction. Similarly, fasting and weight loss have been associated with a decrease of thyroid hormone levels in humans, probably as a result of the decline of circulating leptin (Kok et al., 2005; Reinehr, 2010). In contrast with these findings, in the present study, TT4 serum concentrations unexpectedly significantly increased during caloric restriction, albeit remaining within the range of normality. In humans, the interaction between total calories and carbohydrate intake on thyroid hormone response has been previously investigated. An increase of TT4 was also reported in humans consuming a low-carbohydrates diet, resulting in a decrease of fat mass and an increase in lean mass (Volek et al., 2002). A previous study reported a decrease TT3 and no change in TT4 in response to reduced carbohydrate intake (Mathieson et al., 1986). The significant increase in TT4 observed by Volek et al. (2002) and in the present study, may represent an increase in the biologically active hormone available to cells. However, this speculation should be made with caution since, as in the previously cited study, TT3, free T3, or free T4 were not investigated in the present study.

In the present study, serum concentrations of creatinine decreased during caloric restriction, in accordance with what was previously observed in Beagle dogs during a 17-weeks weight loss study, where only a reduction of body fat with no loss of lean mass was confirmed (Salas-Mani et al., 2018). Serum creatinine concentration is used as a marker of muscle mass in dogs, beyond kidney function, with higher concentration being measured in dogs with great muscle mass, such as sighthound dogs, while lower concentrations are seen in small dogs (Braun et al., 2003; Middleton et al., 2017). In the present study, we did not assess changes in body composition by dual-energy X-ray absorptiometry (DEXA), so that some degree of lean mass loss cannot be completely ruled out. However, considering the slow rate of weight loss that we recorded, and according to what has been observed by other authors (German et al., 2015; German, 2016), it is unlikely that, in this study, obese dogs experienced a significant loss of lean mass during caloric restriction.

In line with our results, obese dogs can show higher serum total protein concentrations than lean dogs (Piantedosi et al., 2016; Radakovich et al., 2017), as a result of decreased serum water fraction, antigenic stimulation, or increased protein catabolism associated to their larger body mass (Radakovich et al., 2017). However, in the present study, we did not observe any differences in the serum concentrations of urea, a biochemical marker of muscle and protein catabolism (Gunst et al., 2013), in OB and CTRL dogs.

### 4.2. Inflammatory status and antioxidant response

Progress in human obesity indicates that adipose tissue plays a major role concerning metabolism and inflammation, and it is involved in the release of inflammatory cytokines influencing systemic inflammatory processes (Wozniak et al., 2009; Stolarczyk, 2017). Advances in canine obesity is still in its early stage; however, a decrease of inflammatory markers, such as CRP and Hp, has been observed after weight loss in previous studies, suggesting that also dogs may suffer from a compelling association between low-grade inflammatory state and obesity (German et al., 2009; Ricci et al., 2011; Wakshlag et al., 2011). Interestingly, experimentally induced overfeeding in laboratory dogs failed to stimulate an increase of inflammatory markers (Tvarijonaviciute et al., 2011; Van de Velde et al., 2013; Moinard et al., 2020). To date, only two studies have explored differences in systemic concentrations of pro-inflammatory markers and cytokines in obese and lean dogs, with a lack of evidence of clear differences (Veiga et al., 2008; Piantedosi et al., 2016). In the present study, we observed higher serum concentrations of acute-phase proteins (CRP and Hp) in obese dogs compared to lean subjects and these findings seem to be in accordance with what has been previously observed in some human studies (Das, 2001; Ste¸pień et al., 2014; Cohen et al., 2021), suggesting that also obese dogs may suffer from a subclinical inflammatory state. However, serum concentrations of pro-inflammatory markers were not affected by caloric restriction in this study, and this finding was in agreement with previous reports in dogs, regardless of the type of obesity, short-term experimentally induced or long-term spontaneous disease (Tvarijonaviciute et al., 2012c; Bastien et al., 2015). This finding may indicate that canine obese-related inflammatory condition is not responsive to changes in energy balance, despite the fact that the degree of weight loss that we observed was in line with the weight loss degrees that are known to improve inflammatory condition in obese humans (Forsythe et al., 2008). Butyrylcholinesterase (BChE) is an enzyme secreted by the liver under the stimulation of free fatty acids flux originating from adipose tissue (Cucuianu et al., 2002), and it is recognized as a robust marker to predict the development and prognosis of low-grade systemic inflammatory conditions, such as obesity, in humans (Das, 2012). In our study, BChE did not differ between obese and lean dogs and was not affected by caloric restriction, in contrast with previous research in canine obesity; in fact, higher BChE activity was assessed in dogs with obesity-related metabolic dysfunction and in those where obesity was induced (Tvarijonaviciute et al., 2010, 2019); moreover, a significant decrease of this enzyme was observed in dogs that experienced rapid, short-term weight loss (Tvarijonaviciute et al., 2013). Contrary to previous findings, however, our study was not conducted in experimental conditions, and the use of antiparasitic collars were not investigated. In fact, some antiparasitic treatments act as acetylcholinesterase inhibitors, and could therefore have affected the levels of BChE in our dogs (Birdane et al., 2022).

Obesity has also been associated with oxidative stress, as a result of an imbalance between oxidant and antioxidant molecules (Sánchez-Rodríguez and Mendoza-Núñez, 2019); moreover, the oxidant components may increase the risk for systemic low-grade chronic inflammation, which may be related to obesity-associated metabolic disorders (Trayhurn, 2005; Donath and Shoelson, 2011; Lumeng and Saltiel, 2011). Interestingly, in our study, OB dogs showed higher markers of total antioxidant capacity (TAC), such as TEACH and Thiol, than lean dogs. A possible interpretation for this finding might be a compensatory system carried out by obese dogs to restore homeostatic balance by enhancing endogenous antioxidants levels, as previously described in obese humans, where a positive correlation between CRP and TAC was observed (Petelin et al., 2017). Interestingly, we observed that TEACH and Thiol were inversely correlated with Hp in obese dogs before the caloric restriction, suggesting that a high degree of low-grade inflammation may result in the partial loss of antioxidant capacity. Nonetheless, the association between antioxidant status and canine obesity remains intriguing and warrants further research.

### 4.3. Fecal bacterial metabolome and microbiota

It is currently well defined that the use of a low-energy, high-protein, high-fiber diet is the most effective strategy to favor weight loss in dogs, reducing voluntary food intake, minimizing muscle loss and attenuating signals of hunger (Blanchard et al., 2004; Laflamme and Hannah, 2005; Weber et al., 2007). The dietary intervention not only helps dogs to lose weight appropriately, but may also impact the gut microbiota and the connection existing between this complex ecosystem and the host; in fact, modulation of the macronutrient content of the diet may affect the intestinal bacteria which in turn may influence the host in many ways, mainly through the release of metabolites that have been acknowledged as beneficial for the host gastrointestinal tract and beyond it.

Among gut microbial metabolites, VFA (mainly acetate, propionate, and butyrate) are produced by anaerobic bacteria in the colon and distal small intestine through the fermentation of resistant starch, dietary fiber, and other low-digestible polysaccharides (Alexander et al., 2019; Ma et al., 2021). In our study, fecal VFA concentrations did not differ between obese and lean dogs and were not affected by dietary treatment. To the best of the author’s knowledge, this is the first study in which fecal VFA concentrations from obese and lean dogs have been compared, while studies in mice and humans have shown higher fecal VFA concentrations among obese than lean individuals (Ley et al., 2006; Schwiertz et al., 2010).

It has to be remembered that different fecal sample collection, preservation, and processing methods may all be significant sources of variation in the quantification VFA in feces (Sowah et al., 2019), and likewise, the rapid absorption rate of VFA by the intestinal mucosa affect their fecal concentrations (Swanson et al., 2002). Nevertheless, the potentially higher production and turnover of VFA in obese mice could be related to differences in microbiota composition and function, with an increased potential for energy harvest among obese (Turnbaugh et al., 2006). The fecal VFA concentrations have been found to be reduced in obese humans after weight loss (Duncan et al., 2007), or unchanged (Damms-Machado et al., 2015); lower VFA fecal concentrations in obese humans during weight-loss may be related to the lower production in response to the lower carbohydrate intake, decreased energy harvest or increased mucosal absorption (Sowah et al., 2019). In line with the reported findings in humans, in a study with obese dogs (Kieler et al., 2017), acetic and propionic acid fecal concentrations were found to be lower in a small group of dogs with fast weight loss rate compared to those with slow weight loss rate.

Biogenic amines include gut microbial metabolites such as cadaverine, putrescine, spermidine, and spermine, and are putrefactive compounds produced by intestinal bacteria from the fermentation of undigested amino acids; biogenic amines are required for cells growth and differentiation, and for the synthesis of DNA, RNA, and proteins (Delzenne et al., 2000). Also, biogenic amines have been correlated in humans with increased fecal odor and increased incidence of colon cancer (Johnson, 1977). In this study, fecal biogenic amines of OB and CTRL dogs were determined, and fecal spermine concentration resulted decreased in CTRL after dietary treatment; it has been previously reported that fecal biogenic amines concentrations may be linked to fecal microbiota (Matsumoto and Benno, 2007). In line with our results, other studies have shown that the diet can impact the fecal biogenic amines concentrations in dogs, as fiber addition decreased fecal proteolysis and biogenic amines production (Jackson and Jewell, 2019), while biogenic amines increased in feces of working dogs fed a grain-free high-protein petfood (Chiofalo et al., 2019). The increase in dietary fiber intake is, therefore, the most likely explanation for the reduction of fecal spermine concentration observed in this study in CTRL dogs after dietary treatment, and, even though not significant, a decrease in fecal spermine concentration was also observed in OB dogs fed the same diet. The effects of spermine on host health remain controversial so far; in fact, spermine seems to possess significative physiological activity and toxicity in mice, being strictly controlled by both colonic microbiota and colonocytes (Matsumoto et al., 2012), but it has also been seen that administration of exogenous spermine inhibited the activation of an inflammatory interleukin in mice, acting as inflammasome inhibitor (Levy et al., 2015). Results from studies conducted with mouse models suggest that biogenic amines metabolism could be dysregulated in the presence of obesity and other metabolic disorders, with the result of impaired glucose regulation, and lipid and energy homeostasis (Ramos-Molina et al., 2019). To date, this is the first study evaluating fecal biogenic amines in obese dogs, and even though their concentrations were affected by neither nutrition status nor caloric restriction, further research is warranted in order to clarify the effects and influence of microbial metabolites in canine obesity.

Studies on the composition of the gut microbiota have provided evidence of existing differences in the bacterial taxa found in lean and obese dogs, with sometimes controversial results (Handl et al., 2013; Park et al., 2015; Forster et al., 2018). At the phylum level, Bacteroidetes (range of abundance 12–38%) together with Firmicutes (14%–48%) and Fusobacteria (7%–44%) co-dominate the core bacterial community both in healthy and obese dogs (Middelbos et al., 2010; Suchodolski, 2011; Herstad et al., 2017; Salas-Mani et al., 2018). In this study, Firmicutes were the dominant phylum in obese as well as in lean dogs, as previously reported in a study involving pet dogs (Handl et al., 2013). However, when obesity was induced in dogs, the proportion of Proteobacteria was increased and Firmicutes were decreased, with the result of Proteobacteria being the dominant phylum in the obese animals (Park et al., 2015). In the present study, we did not observe any difference in the proportions of the major phyla between obese and lean dogs; conversely, inconsistently with our results, two previous researches showed that Actinobacteria were more abundant in obese than in lean pet dogs (Handl et al., 2013; Forster et al., 2018).

In obesity studies involving humans, mice and dogs, differences detected in Bacteroidetes and Firmicutes abundances are widely used as metric of impaired intestinal homeostasis, and an increased Firmicutes/Bacteroidetes (F/B) ratio is usually associated with obesity in mice (Ley et al., 2005) and humans (Ley et al., 2006; Kasai et al., 2015; Koliada et al., 2017). However, other studies revealed no significant differences in the F/B ratio between lean and obese humans (Ismail et al., 2011; Hu et al., 2015) or even opposite findings (Schwiertz et al., 2010; Vaiserman et al., 2020), so that it can be supposed that differences in other phyla, host age and sex, as well as environmental and genetic factors may also affect the F/B ratio (Stojanov et al., 2020; Vaiserman et al., 2020).

In this study, the dietary treatment had a significant impact on the fecal microbiota composition of OB dogs, mainly within the predominant phyla Firmicutes and Bacteroidetes. For instance, the fecal abundance of *Bacteroides* spp. increased, and Firmicutes decreased, in obese in response to the diet fed at maintenance requirement, while no difference was seen between obese and lean dogs; this finding appears to be in agreement with what was observed in a previous study that aimed to evaluate the effects of diets dissimilar in macronutrient composition on dogs of diverse body conditions, finding no differences in bacterial abundances among groups (Li et al., 2017).

The genus *Bacteroides* consists of bile-tolerant microorganisms associated with the consumption of diets rich in protein and fat in humans and dogs (De Filippo et al., 2010; Wu et al., 2011; Salas-Mani et al., 2018), although *Bacteroides* in the latter species are more selective for protein-rich substrates, as a reduction in the fecal abundance of *Bacteroides* occurred in response to the increase of dietary fat in dogs fed high-fat diets (>30% on a dry matter basis; Kilburn et al., 2020). In line with our results, *Bacteroides* were significantly increased in the feces of six healthy dogs fed for 21 days a weight loss diet (Mori et al., 2019) that had a macronutrient profile (carbohydrate, fat and protein content) comparable to the diet that was used in the present study (with the exception of the total dietary fiber content, 28% in the study by Mori et al. vs. 17% in our study, as fed).

*Bacteroides* has been negatively correlated with energy intake and adiposity in humans and dogs (Yatsunenko et al., 2012; Kovatcheva-Datchary and Arora, 2013; Bermudez Sanchez et al., 2020) and the administration of *B. uniformis* CECT7771 was able to reduce weight gain and serum triglycerides and cholesterol concentrations in mice fed high-fat diets (Gauffin Cano et al., 2012).

In this study, Firmicutes, which include species known to metabolize dietary plant polysaccharides and produce VFA (Pilla and Suchodolski, 2021), significantly decreased in the feces of OB dogs after dietary treatment. Consistently with our results, in a previous study (Mori et al., 2019), the proportion of Firmicutes as well as F/B ratio in feces of healthy dogs were lower when animals were fed a weight loss diet than when they received diets proposed for other diseases. Similar findings were observed in obese humans after weight reduction, where a lower F/B ratio was defined as restitution to “lean phenotype” (Ley et al., 2006; Clemente et al., 2012). In our study, the F/B ratio was not significantly affected, but the dietary treatment induced a shift of bacterial taxa considered of key significance in relation to obesity, such as those belonging to the phylum Firmicutes as well as to the family *Bacteroides*. These findings may suggest that feeding a high-protein, high-fiber diet may play a role in the modulation of fecal microbiota in canine obesity. Conversely, in the present study, Firmicutes and Bacteroidetes were not affected by diet in lean dogs. It has been seen that the composition of intestinal microbiota in healthy dogs can be unaffected (Bresciani et al., 2018) or be effectively modified (Mori et al., 2019) by short-term dietary interventions. The effectiveness of persistent changes in the intestinal microbiota induced by dietary interventions has been associated, in recent studies, to prolonged experimental set-ups, as well as to the shift to diets that were extremely different in terms of macronutrients composition (Mori et al., 2019; Allaway et al., 2020). However, obese dogs do not seem to show a clear state of intestinal dysbiosis: in fact, in the present study, the DI of obese dogs remained within the established reference interval for healthy dogs both before and after caloric restriction, in accordance with results previously reported by other authors (Bermudez Sanchez et al., 2020; Phungviwatnikul et al., 2022). Similarly, a relative abundance of microbial populations, as well as diversity of the microbial community, did not differ between OB and CTRL, in accordance with results from a previous study involving a number of lean and obese colony dogs similar to ours (Handl et al., 2013); on the contrary, other authors have recently reported lower microbial diversity in obese dogs in comparison to lean dogs; in particular, the results described by Park et al. (2015) derived from a lower number of dogs compared to ours, while in the study by Forster et al. (2018) 66 animals, among obese and lean dogs, have been evaluated.

The 16S rRNA gene profiling revealed that OB dogs had a lower abundance of Erysipelotrichi, Erysipelotrichales, Erysipelotrichaceae, *Eubacterium* and *E. biforme* (data not shown for class and order), compared with CTRL dogs. The same findings were observed in a previous study which enrolled pet dogs and compared microbial abundances in obese and normal weight dogs (Forster et al., 2018). Hence, Erysipelotrichaceae and *Eubacterium* spp. abundance seem to be negatively correlated with obesity in dogs, while a positive correlation has been described in humans, where high levels of *E. dolichum* have been associated to increased visceral fat mass (Pallister et al., 2017; Pinart et al., 2022). *Eubacterium* spp*.,* one of the core genera of the human gut microbiota, belongs to Firmicutes and consists of bacterial taxa that have been shown to be involved in carbohydrate metabolism and degradation of dietary fiber; in fact, increased levels of *Eubacterium* spp. are associated with the production of organic acids, from carbohydrates or peptone, including butyric, acetic and formic acids (Louis and Flint, 2017; Mukherjee et al., 2020). In a study by Forster et al. (2018), obese dogs were characterized by a lower abundance of *Eubacterium* spp. and *E. biforme* and reduced concentrations of VFA. In fact, the abundance of *Eubacterium* spp. and other butyrate-producing bacteria in the gut is strongly correlated with VFA concentrations, and the ingestion of dietary fibers has been seen to increase VFA concentrations and abundance of *Eubacterium* spp. (Duncan et al., 2007). However, in the current study, fecal VFA concentrations were not affected, suggesting that the relationship between the fecal abundance of *Eubacterium* spp. and VFA concentrations in obese dogs needs further investigation. In a study with elderly humans, the abundance of *Eubacterium* spp. was negatively correlated with CRP (Ghosh et al., 2020); in accordance with this finding, in the present study, at T0, CTRL dogs showed higher abundance of *Eubacterium* spp. and lower concentrations of inflammatory markers (CRP and Hp), compared with OB.

Erysipelotrichaceae consists of a bacterial family that has been identified in the fecal microbiota of healthy dogs (Garcia-Mazcorro et al., 2012a; Gagné et al., 2013; Panasevich et al., 2014). Interestingly, members of this family have been shown to change in abundance in response to changes in dietary macronutrient composition; in a study performed with dogs fed either kibbles or a raw-meat based diet, Erysipelotrichaceae were positively correlated with dietary fat content and markers linked to carbohydrate fermentation (such as VFA) on one hand, and negatively correlated with crude protein content of the diet on the other (Bermingham et al., 2017); moreover, Erysipelotrichaceae seem to be affected neither by type of protein nor by fat sources (Panasevich et al., 2014). One possible explanation for the decrease in Erysipelotrichaceae abundance that we observed in lean dogs after the dietary treatment may therefore be associated with the high protein content of the weight loss diet. Similarly, low levels of *Eubacterium* spp. have been associated with increased protein, fat, and fructose intake in humans and the consumption of sugar-rich diets in mice (Duncan et al., 2007; Mahowald et al., 2009; Wu et al., 2011; Jones et al., 2019).

An expected result of weight reduction in humans is an increase in the diversity of bacterial communities. However, conflicting results have been obtained depending on the method by which the weight loss was achieved (Damms-Machado et al., 2015; Remely et al., 2015). In a previous study, a reduction of Bacteroidetes abundance was observed in human patients that underwent obesity surgery, while, on the contrary, an increase of this phylum was seen secondary to treatment with a low-calorie diet (Damms-Machado et al., 2015). Other studies found no alteration of Bacteroidetes after weight reduction, challenging the reversibility of reduced Bacteroidetes abundance in obese humans (Remely et al., 2015; Frost et al., 2019).

In the present study, the caloric restriction did not have a significant impact on bacterial diversity and this finding is consistent with the results from a previous research, in which the fecal microbiota of 6 obese Beagle dogs was not affected by a 17-weeks weight loss program (Schauf et al., 2018). Conversely, in a recent study, 20 obese pet dogs showed an increase in bacterial diversity when they reached their ideal body weight; in the same study, bacterial diversity was not improved in a small group of dogs with less effective weight loss (Bermudez Sanchez et al., 2020). In the study by Bermudez Sanchez et al. (2020), the phylum Bacteroidetes significantly increased after weight loss in dogs, and this increase was driven mainly by *Bacteroides* spp.; conversely, in the present study, a decrease in the fecal abundance of this genus was observed after 180 days of caloric restriction. However, in the study by Bermudez Sanchez et al. (2020), dogs were followed until they reached the ideal weight (mean duration = 330 days), whereas our study lasted 180 days and only 3 dogs out of 16 had reached their ideal weight at the end of the trial.

*Collinsella* spp. belong to the Actinobacteria phylum and have been described as fiber degraders and H_2_ consumers, resulting in the production of mainly lactate and acetate. *Collinsella* spp. and *C. aerofaciens* have been proposed as biomarkers of obesity in humans, as they are positively associated with body mass index and insulin resistance (Companys et al., 2021); moreover, it has been seen that abundance of *Collinsella* spp. in humans was significantly reduced during a weight loss program (Frost et al., 2019; Martínez-Cuesta et al., 2021). In the present study, abundance of *Collinsella* spp. and *C. stercoris* numerically decreased after 90 days of caloric restriction and then significantly increased at the end of the study compared with the previous time point. According to our findings, several human studies have previously shown that changes in the diversity and composition of the gut microbiome rapidly occur during dietary intervention (e.g., caloric reduction) or obesity surgery; nevertheless, these changes are only partially sustained over time, tending toward a regression to baseline, irrespective of the weight loss achieved (Simões et al., 2014; Heinsen et al., 2017; Frost et al., 2019; Shen et al., 2019). On the contrary, in this study, the family Clostridiaceae was consistently associated with weight loss, increasing its abundance at the end of the study compared with trial start. To date, results on Clostridiaceae abundance in obese dogs are not univocal, and the degree of caloric reduction may represent an important factor; in fact, in previous studies with obese dogs, the genus *Clostridium* showed a decrease after a weight loss program (Salas-Mani et al., 2018; Bermudez Sanchez et al., 2020), in line with what had been observed in humans (Nadal et al., 2009). On the contrary, in other studies with dogs, the use of a high-protein weight-loss diet, fed without any caloric restriction, increased Clostridiaceae (Zentek et al., 2004; Li et al., 2017; Mori et al., 2019), suggesting that Clostridiaceae may be stimulated by dietary protein, rather than by dietary fiber, as previously reported (Panasevich et al., 2013). Our results seem to suggest that the increase in Clostriadaceae was caused by caloric restriction, rather than by the diet, because no changes in their abundance were observed during the first phase of the study.

The study has some limitations. The use of private-owned animals, rather than research dogs introduced variables, both dogs and owners related. Factors affecting populations variability included signalment and different environmental conditions. In addition, the small study populations might not be able to show some significant differences between compared groups. However, results from the present study are plausibly more representative of the overall canine population. A second limitation was related to ethical limitations: in fact, given the requirement for sedation, DEXA scanning cannot be performed in obese dogs, therefore the changes of lean mass or fat mass, before and after the weight loss, were not assessed. Finally, it should be also noted that fT4 was not measured neither the use of antiparasitic collars were investigated in obese dogs, therefore only careful conclusions have been formed regarding thyroid homeostasis and BChE activity in obese dogs.

## 5. Conclusion

The present study has provided evidence that obese dogs suffer from a subclinical inflammatory state, characterized by higher levels of some inflammatory markers and a concomitant higher total antioxidant capacity. However, caloric restriction did not influence the inflammatory status of obese dogs.

The fecal microbiota of obese and lean dogs did not display big differences and neither bacterial diversity nor metabolites (VFA and polyamines) were influenced by dogs’ nutritional status.

Caloric restriction resulted in a few changes in the abundance of some bacterial populations but failed to affect bacterial diversity, DI, and metabolites in obese dogs. However, the decrease of *Bacteroides* spp. and the increase of Clostriadiaceae family, were the only changes consistently associated with caloric restriction and weight loss throughout the study.

This study has provided insights into the involvement of the intestinal microbiota, inflammatory and antioxidant status as well as thyroid homeostasis in canine obesity. Further research is warranted to better clarify the influence of these factors on canine obesity, since the mechanism of these connections is somewhat far from being conclusive.

## Data availability statement

The datasets presented in this study can be found in online repositories. The names of the repository/repositories and accession number(s) can be found at: NCBI Sequence Read Archive under accession number PRJNA822358.

## Ethics statement

The animal study was reviewed and approved by Animal Welfare Committee of the University of Bologna (number 96551/2019). Written informed consent was obtained from the owners for the participation of their animals in this study.

## Author contributions

FF and GB conceived and designed the study. CV, SG, and ED carried out the clinical study and collected all data. CV, CP, RP, AT, CR, ED, CD, and CS carried out laboratory work. CV and RP performed statistical analyses. CV wrote the original draft with assistance and feedback from SG, CP, RP, JS, AT, CR, CS, EP, FF, and GB. All authors contributed to the article and approved the submitted version.

## Funding

This study received funding from Monge & C. S.p.A., Monasterolo di Savigliano, Italy (grant number 0018554/2019).

## Conflict of interest

EP is an employee of Monge & C S.p.A, Monasterolo di Savigliano, Italy. JS and RP were employed of the GI Lab at Texas A&M University, which provides the dysbiosis index on a fee for service basis.

The remaining authors declare that the research was conducted in the absence of any commercial or financial relationships that could be construed as a potential conflict of interest.

The authors declare that this study received funding from Monge & C S.p.A. The funder had the following involvement in the study: assistance during manuscript preparation.

The reviewer CWC declared a shared affiliation with the authors RP, JS to the handling editor at the time of review.

## Publisher’s note

All claims expressed in this article are solely those of the authors and do not necessarily represent those of their affiliated organizations, or those of the publisher, the editors and the reviewers. Any product that may be evaluated in this article, or claim that may be made by its manufacturer, is not guaranteed or endorsed by the publisher.
