# Supplementary material for: Fecal microbiota and inflammatory and antioxidant status of obese and lean dogs, and the effect of caloric restriction
Source: Front Microbiol. 2023 Jan 12;13:1050474. doi: 10.3389/fmicb.2022.1050474 (PMC9878458; doi:10.3389/fmicb.2022.1050474)
Supplement: Supplementary file 2 [file Table_2.docx]

Supplementary Material

Fecal microbiota and inflammatory and antioxidant status of obese and lean dogs, and the effect of caloric restriction

Carla Giuditta Vecchiato*, Stefania Golinelli, Carlo Pinna, Rachel Pilla, Jan S. Suchodolski, Asta Tvarijonaviciute, Camila Peres Rubio, Elisa Dorato, Costanza Delsante, Claudio Stefanelli, Elena Pagani, Federico Fracassi and Giacomo Biagi

Correspondence *: Carla Giuditta Vecchiato: carla.vecchiato2@unibo.it

# Supplementary Tables

## Supplementary Table 1

Baseline characteristics of study dogs: obese dogs (OB, n=16) and lean dogs (CTRL, n=15)

|  | **OB T0** | **CTRL T0** |
| --- | --- | --- |
| Sex | 5 M; 5 NM; 2 F; 4 NF | 4 M; 6 NM; 5 NF |
| Age (months) | 66 [20-111] | 72 [14-116] |
| Breed | AS 1, Beagle 1, BMD 2, Chihuahua 1, EB 1, GR 1, GS 2, LR 1, MB 5, Pug 1 | BC 3, GR 1, GS 2, JRT 1, LR 2, MB 6, |
| Daily energy intake^a^ | 103 [87-186] | 199 [70-187] |

F, female; NF, neutered female; M, male; NM, Neutered male.

Breed acronyms: AS, Australian shepherd; BC Border collie; BMD, Bernese mountain dog; EB, English bulldog; GR, Golden retriever; GS, German shepherd; JRT, Jack Russel Terrier; LR, Labrador retriever; MB mixed-breed.

^a^ Expressed as kcal of ME per kg^0.75^ of target body weight (obese dogs) /current body weight (lean dogs)

## Supplementary Table 2

Fecal abundance (Log DNA) of selected bacterial populations of obese (OB, n=16) and lean (CTRL, n=15) dogs fed the same experimental diet for 30 days (phase 1 of the study), based on qPCR.

|  | OB | | CTRL | |  | ANOVA *p*-value | | |
| --- | --- | --- | --- | --- | --- | --- | --- | --- |
|  | T0 | T30 | T0 | T30 | SEM | Group | Diet | Group × Diet |
| *Faecalibacterium* spp.  RI [3.4-8.0] | 5.21 | 5.51 | 6.08 | 6.44 | 0.199 | 0.024 | 0.400 | 0.939 |
| *Turicibacter* spp.  RI [4.6-8.1] | 6.84 | 7.03 | 7.38 | 7.25 | 0.133 | 0.173 | 0.878 | 0.595 |
| *Streptococcus* spp.  RI [1.9-8.0] | 5.70 | 4.80 | 5.34 | 4.77 | 0.182 | 0.591 | 0.045 | 0.660 |
| *E. coli*  RI [0.9-8.0] | 5.21 | 4.92 | 5.12 | 5.12 | 1.817 | 0.902 | 0.770 | 0.756 |
| *Blautia* spp.  RI [9.5-11] | 10.8 | 10.7 | 10.9 | 10.9 | 0.034 | 0.058 | 0.397 | 0.466 |
| *Fusobacterium* spp.  RI [7.0-10.3] | 9.48 | 9.81 | 9.44 | 9.82 | 0.553 | 0.909 | 0.013 | 0.853 |
| *C. hiranonis*  RI [5.1-7.1] | 6.49 | 6.58 | 6.58 | 6.85 | 0.097 | 0.360 | 0.349 | 0.654 |

OB: obese dogs; CTRL: lean dogs.

SEM: standard error of the mean.

RI: reference intervals established based on 120 healthy dogs.

## Supplementary Table 3

Levels of serum metabolites (mean ± SD or median [range]) in obese and lean dogs fed the same experimental diet for 30 days (phase 1 of the study).

| **parameter** | **OB T30** | **CTRL** | ***p*-value** |
| --- | --- | --- | --- |
| Glycemia (mg/dl)  RI [65-115] | 91 ± 8.36 | 87 ± 9.75 | 0.175 |
| Creatinine (mg/dl)  RI [0.75-1.4] | 1.16 ± 0.13 | 1.16 ± 0.2 | 0.943 |
| Urea (mg/dl)  RI [17-48] | 41 ± 6.62 | 44 ± 12.9 | 0.334 |
| AST (U/L)  RI [15-52] | 33 ± 6.44 | 35 ± 7.68 | 0.561 |
| ALT (U/L)  RI [15-65] | 43 [19-128] | 44 [26-142] | 0.463 |
| ALP (U/L)  RI [12-180] | 35 [20-146] | 35 [15-150] | 0.733 |
| P (mg/dl)  RI [2.65-5.40] | 3.46 ± 0.79 | 4.23 ± 0.73 | **0.008** |
| Albumin (g/dl)  RI [2.75-3.85] | 3.27 ± 0.24 | 3.21 ± 0.17 | 0.418 |
| Total protein (g/dl)  RI [5.60-7.30] | 6.78 ± 0.44 | 6.45 ± 0.37 | **0.033** |
| Na (mEq/L)  RI [143-151] | 147 ± 1.46 | 147 ± 1.94 | 0.634 |
| K (mEq/L)  RI [3.80-5.0] | 4.4 [4-4.9] | 4.6 [4-4.9] | 0.340 |
| GGT (U/L)  RI [0-5.0] | 2.5 [1.1-3.8] | 1.9 [1.1-5.4] | 0.488 |
| Cholesterol (mg/dl)  RI [123-345] | 240 ± 51 | 264 ± 74 | 0.307 |
| Triglycerides (mg/dl)  RI [30-120] | 70 ± 25 | 34 ± 35 | 0.975 |
| TT4 (nmol/L)  RI [13-51] | 23 ± 9.2 | 17 ± 9.7 | 0.07 |
| TSH (ng/ml)  RI [0.03-0.38] | 0.17 [0.1-0.5] | 0.15 [0.1-0.5] | 1 |

OB: obese dogs; CTRL: lean dogs.

RI: reference interval.

ALP: alkaline phosphatases; ALT: alanine aminotransferase; AST: aspartate aminotransferase; GGT: gamma-glutamyl transferase; TSH: thyroid stimulating hormone; TT4: total thyroxine.

## Supplementary Table 4

Fecal abundance (Log DNA) of selected bacterial populations of obese dogs (n=16) before (T30), after 90 days (T120) and at the end (T210) of caloric restriction (phase 2 of the study), based on qPCR.

|  | T30 | T120 | T210 | Pooled SEM | *p*-value |
| --- | --- | --- | --- | --- | --- |
| *Faecalibacterium* spp.  RI [3.4-8.0] | 5.51 | 6.42 | 6.47 | 0.271 | 0.029 |
| *Turicibacter* spp.  RI [4.6-8.1] | 7.03 | 6.54 | 6.69 | 0.238 | 0.348 |
| *Streptococcus* spp.  RI [1.9-8.0] | 4.80 | 5.14 | 5.23 | 0.265 | 0.491 |
| *E. coli*  RI [0.9-8.0] | 4.92 | 3.62 | 4.00 | 0.382 | 0.062 |
| *Blautia* spp.  RI [9.5-11] | 10.7 | 10.8 | 10.9 | 0.046 | 0.071 |
| *Fusobacterium* spp.  RI [7.0-10.3] | 9.80 | 10.0 | 9.84 | 0.103 | 0.315 |
| *C. hiranonis*  RI [5.1-7.1] | 6.58 | 6.84 | 6.85 | 0.139 | 0.315 |

SEM: standard error of the mean.

## Supplementary Table 5

Levels of serum metabolites (mean ± SD or median [range]) in obese dogs before (T30), after 90 days (T120) and at the end (T210) of caloric reduction (phase 2 of the study).

| parameter | T30 | T120 | T210 | *p*-value |
| --- | --- | --- | --- | --- |
| Glycemia (mg/dl)  RI [65-115] | 90 [81-112] | 101 [58-119] | 101 [78-112] | 0.292 |
| Creatinine (mg/dl)  RI [0.75-1.4] | 1.16 ± 0.13^a^ | 1.06 ± 0.13^b^ | 1.02 ± 0.13^b^ | **<0.0001** |
| Urea (mg/dl)  RI [17-48] | 41 ± 6.62 | 43 ± 8.68 | 40 ± 11.9 | 2.116 |
| AST (U/L)  RI [15-52] | 34 [22-45] | 30 [18-87] | 31 [22-46] | 0.072 |
| ALT (U/L)  RI [15-65] | 43 [19-128] | 38 [22-90] | 44 [23-82] | 0.285 |
| ALP (U/L)  RI [12-180] | 35 [20-146] | 30 [17-148] | 32 [14-136] | 0.362 |
| Albumin (g/dl)  RI [2.75-3.85] | 3.27 ± 0.24 | 3.20 ± 0.27 | 3.29 ± 0.25 | 0.106 |
| Total protein (g/dl)  RI [5.60-7.30] | 6.78 ± 0.44^a^ | 6.29 ± 0.55^b^ | 6.71 ± 0.38^a^ | **<0.0001** |
| GGT (U/L)  RI [0-5.0] | 2.5 ± 0.88^a^ | 3.30 ± 0.88^b^ | 2.69 ± 0.84^a^ | **0.009** |
| Cholesterol (mg/dl)  RI [123-345] | 240 [151-341] | 224 [140-395] | 222 [145-450] | 0.570 |
| Triglycerides (mg/dl)  RI [30-120] | 70 ± 25 | 62 ± 19 | 76 ± 26 | 0.202 |
| TT4 (nmol/L)  RI [13-51] | 23 ± 9.2^a^ | 33 ± 10^b^ | 33 ± 15^b^ | **0.007** |
| TSH (ng/ml)  RI [0.03-0.38] | 0.17 [0.1-0.5] | 0.20 [0.1-0.3] | 0.18 [0.04-0.3] | 0.144 |

RI: reference interval.

ALP: alkaline phosphatases; ALT: alanine aminotransferase; AST: aspartate aminotransferase; GGT: gamma-glutamyl transferase; TSH: thyroid stimulating hormone; TT4: total thyroxine.

Values with different superscripts are significantly different in multiple comparisons with FDR adjustment.
